# The complete chloroplast genome of *Camellia gauchowensis* and its phylogenetic analysis

**DOI:** 10.1080/23802359.2020.1772694

**Published:** 2020-06-02

**Authors:** Yuanlan Zhang, Guibin Wang

**Affiliations:** a The Southern Modern Forestry Collaborative Innovation Center, Nanjing Forestry University, Nanjing, Jiangsu, China; b College of Forestry, Nanjing Forestry University, Nanjing, Jiangsu, China

**Keywords:** *Camellia gauchowensis*, chloroplast genome, phylogenetic analysis

## Abstract

*Camellia gauchowensis* is an economic woody edible oil tree species with high yield per unit area and high ornamental value, which is commonly cultivated in the south of China. The complete chloroplast (cp) genome of *C. gauchowensis* was assembled and annotated based on the Illumina pair-end sequencing. The whole cp genome of *C. gauchowensis* is 157,004 bp in size and comprises of a large single-copy (LSC) region of 86,657 bp and a small single-copy (SSC) region of 18,297 bp separated by a pair of inverted repeat (IR) regions of 26,025 bp each. It encodes a total of 129 genes, including 81 protein-coding genes, 5 ribosomal RNA genes, and 43 transfer RNA genes. The neighbor-joining phylogenetic analysis shows that *C. gauchowensis* is evolutionarily closest to *C. cuspidata*.

*Camellia gauchowensis* belongs to the genus *Camellia* of Theaceaen, which is an oil crop growing specifically in south China. It has been cultivated for more than 2300 years and plays an important role in Chinese forestry (Zhang et al. 2018). Although it has economic and horticultural importance, there are a few researches on *C. gauchowensis* at present. Here, we assembled and annotated the complete cp genome of *C. gauchowensis* through high-throughput sequcencing, and then submitted the genome sequence with gene annotations to GenBank (NCBI Accession Number: MT449927). Also, a phylogenetic analysis of *C. gauchowensis* was performed. These studies would be helpful for its molecular phylogenetic and genetic diversity analyses in future.

Genomic DNA was extracted from the fresh leaves of *C. gauchowensis* collected from Jinhua International Camellia Species Park (Zhejiang Province, China; Coordinates: 29°7′10.1208″N, 119°35′52.1088″E). The specimen (Accession number: 2020_cg) was deposited in the Key Laboratory of Forest Genetics and Biotechnology, Ministry of Education, Nanjing Forestry University. Total genomic DNA was extracted with the MiniBEST plant Genomic DNA Extraction Kit (Takara, Dalian, China), and paired-end (PE150) sequencing was performed on the Illumina Hiseq 2500 platform (Illumina, San Diego, CA, USA). We gained 28,424,252 raw reads, and 27,343,519 clean reads left after quality control by Trimmomatic (Wang et al. 2020). Then, the clean reads were aligned to the reference genome *C. japonica* (NCBI Accession Number: NC_036830.1) through Bowtie2 (Wang et al. 2018; Cao et al. 2020) and the cp genome of *C. gauchowensis* was assembled by Fast-Plast v1.2.8. The assembled cp genome was annotated through the online annotation program DOGMA (Wyman et al. 2004) with a manual check.

The complete cp genome of *C. gauchowensis* is 157,004 bp in size with a typical quadripartite structure, containing a large single-copy (LSC) region of 86,657 bp and a small single-copy (SSC) region of 18,297 bp separated by a pair of inverted repeat (IR) regions of 26,025 bp. The total GC content is 37.29%, while the corresponding values of the LSC, IR, and SSC regions are 35.29%, 42.98%, and 30.55%, respectively. The cp genome possesses 129 functional genes, including 81 protein-coding genes, 5 ribosomal RNA genes, and 43 transfer RNA genes.

Phylogenetic analysis was performed based on the complete cp genomes of *C. gauchowensis* and other 10 *Camellia* species. The evolutionary history was inferred by neighbour-joining method in MEGA v7.0.14 (Kumar et al. 2016). As illustrated in Figure 1, the genetic relationship between *C. gauchowensis* and *C. cuspidata* (NC_022459.1) was found to be closest.

**Figure 1.**
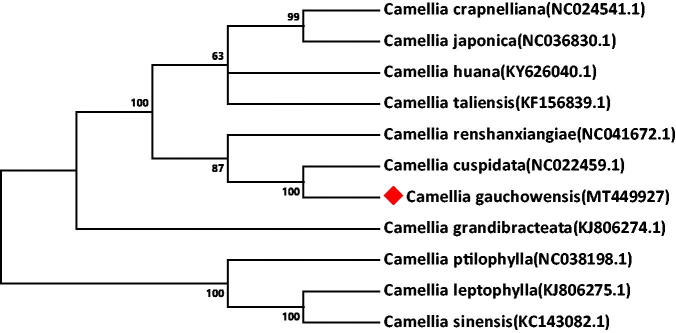
The neighbor-joining phylogenetic tree of 11 *Camellia* cp genomes were conducted with MEGA v7.0.14. The bootstrap values from 1000 replicates are listed for each node.

## Data Availability

The data that support the findings of this study are openly available in GenBank of NCBI at https://www.ncbi.nlm.nih.gov, reference number MT449927, or available from the corresponding author.
